# Fc-Mediated E2-Dimer Subunit Vaccines of Atypical Porcine Pestivirus Induce Efficient Humoral and Cellular Immune Responses in Piglets

**DOI:** 10.3390/v13122443

**Published:** 2021-12-06

**Authors:** Xujiao Ren, Ping Qian, Shudan Liu, Huanchun Chen, Xiangmin Li

**Affiliations:** 1 State Key Laboratory of Agricultural Microbiology, Huazhong Agricultural University, Wuhan 430070, China; renxujiao92@163.com (X.R.); qianp@mail.hzau.edu.cn (P.Q.); vet2439134082@163.com (S.L.); chenhch@mail.hzau.edu.cn (H.C.); 2 Laboratory of Animal Virology, College of Veterinary Medicine, Huazhong Agricultural University, Wuhan 430070, China; 3 Key Laboratory of Preventive Veterinary Medicine in Hubei Province, The Cooperative Innovation Center for Sustainable Pig Production, Wuhan 430070, China

**Keywords:** APPV, congenital tremor, subunit vaccine, Fc fragment, dimer

## Abstract

Congenital tremor (CT) type A-II in piglets is caused by an emerging atypical porcine pestivirus (APPV), which is prevalent in swine herds and a serious threat to the pig production industry. This study aimed to construct APPV E2 subunit vaccines fused with Fc fragments and evaluate their immunogenicity in piglets. Here, APPV E2Fc and E2ΔFc fusion proteins expressed in *Drosophila* Schneider 2 (S2) cells were demonstrated to form stable dimers in SDS-PAGE and western blotting assays. Functional analysis revealed that aE2Fc and aE2ΔFc fusion proteins could bind to FcγRI on antigen-presenting cells (APCs), with the affinity of aE2Fc to FcγRI being higher than that of aE2ΔFc. Moreover, subunit vaccines based on aE2, aE2Fc, and aE2ΔFc fusion proteins were prepared, and their immunogenicity was evaluated in piglets. The results showed that the Fc fusion proteins emulsified with the ISA 201VG adjuvant elicited stronger humoral and cellular immune responses than the IMS 1313VG adjuvant. These findings suggest that APPV E2 subunit vaccines fused with Fc fragments may be a promising vaccine candidate against APPV.

## 1. Introduction

Congenital tremor (CT) in piglets, commonly known as “shaking pig disease”, is a neurologic disease that occurs in newborn piglets. Affected piglets show clinical symptoms, including rhythmic tremors in the limbs and head and paroxysmal spasm, further complicated by ataxia. This condition results in decreased suckling ability of piglets, leading to starvation due to insufficient colostrum intake and eventual death. CT syndrome was first described in the United States in 1922; however, the causative agent was initially unknown [1]. Since then, the disease has been reported in some European and Asian countries, suggesting its global distribution [2]. CT can be classified into different subtypes according to etiology and whether the central nervous system (CNS) has histopathological lesions. Specifically, CT type A-II, a disease prevalent in the pig breeding industry, has been proven to be caused by a newly emerging virus called atypical porcine pestivirus (APPV) [3,4]. Interestingly, other viruses, including astrovirus, lateral-shaking inducing neurodegenerative agent (LINDA) virus, porcine circovirus-like virus P1, and porcine teschovirus (PTV) [5,6,7,8] have also been detected in CT-affected piglets by metagenomic sequencing, but only APPV has been confirmed worldwide and has been proven to fulfill Koch’s postulates [2,3,4].

APPV is a member of the genus *Pestivirus* of the family *Flaviviridae* [9]. The APPV genome is a positive-sense single-stranded RNA (+ssRNA) with a length of approximately 11.5 kb, containing a single open reading frame (ORF) flanked by a 5′-untranslated region (5′-UTR) and 3′-untranslated region (3′-UTR). The ORF of APPV is composed of 3635 amino acids (aa), which encodes a polyprotein precursor that is putatively processed into four structural proteins (C, E^rns^, E1, and E2) and eight non-structural proteins (N^pro^, P7, NS2, NS3, NS4A, NS4B, NS5A, and NS5B) [9,10,11,12,13]. The E2 protein is the main antigenic protein in pestiviruses, which can induce the production of protective neutralizing antibodies and thus, become the main target for the development of subunit vaccines [14,15,16].

In recent years, APPV-infected CT type A-II has become prevalent in swine herds [2,3,4,7,17,18,19,20], which seriously threatens the healthy development of pig production. Currently, no commercial vaccines or antiviral drugs are available for APPV infections. Moreover, the inability to effectively obtain live virus particles severely hampers the development of APPV vaccines. At present, only two studies on APPV vaccines have been reported. One is the APPV E2 subunit vaccine prepared in our laboratory using a baculovirus expression system and was demonstrated to induce a Th2-type immune response in mice [14]. The other is a recently published study on the virus-like particle vaccine, which showed that virus-like particles, based on the self-assembly of E^rns^ and E2 proteins, can induce a strong antibody response and reduce the viral load in the tissues of BALB/c mice [15]. These studies suggest that E2 protein can be developed as a safe and effective subunit vaccine against APPV infection, but its immunogenicity in pigs remains to be further explored.

The IgGFc fragment can be used as a vaccine molecular adjuvant to significantly stimulate mucosal, cellular, and humoral immune responses in piglets [16]. Additionally, accumulating evidence has indicated that the Fc fusion protein facilitates the dimerization of the protein through the disulfide bond of the Fc hinge region, which increases the stability and half-life of the protein [21,22,23,24]. Hence, as an effective molecular adjuvant, the IgGFc fragment might be of great value in the development of potential APPV vaccines.

Here, three recombinant *Drosophila* Schneider 2 (S2) cell lines expressing APPV E2, E2Fc, and E2ΔFc fusion proteins were constructed to evaluate the immunogenicity of the fusion proteins in piglets. The aE2ΔFc fusion protein emulsified with the ISA 201VG adjuvant induced more effective humoral and cellular immune responses in piglets, suggesting that APPV E2 subunit vaccines fused with Fc fragments may be a promising vaccine candidate against APPV infection.

## 2. Materials and Methods

### 2.1. Construction of Recombinant S2 Cell Lines

The pMT-Bip-V5-HisA vectors expressing APPV E2, E2Fc, and E2ΔFc fusion proteins were constructed. The pEASY-Blunt-APPV E2 plasmid containing the APPV E2 gene (GenBank No.KY652092.1) without the endogenous transmembrane domain was maintained in our laboratory [18]. The swine IgG3Fc fragment containing the hinge region, an extended CH2, and a CH3 domain (GenBank No.AK405781.1) [16] was synthesized. Furthermore, an IgG3ΔFc fragment retaining the dimer interface and receptor binding sites was identified using conserved domain analysis (https://www.ncbi.nlm.nih.gov/Structure/cdd/wrpsb.cgi (accessed on 25 July 2019). Then, APPV E2, E2Fc, and E2ΔFc fragments were amplified by PCR or overlap PCR using the primers shown in Table S1. Finally, the three PCR products were inserted into the pMT-Bip-V5-HisA vector (kindly provided by Professor Yu Guo, College of Pharmacy, Nankai University), including a *Drosophila* BiP signal sequence, yielding the expression vectors pMT-Bip-aE2, pMT-Bip-aE2Fc, and pMT-Bip-aE2ΔFc. Expression vectors were verified by restriction analysis and sequencing.

Wild-type S2 cells were kindly provided by Professor Yu Guo from the College of Pharmacy, Nankai University. The S2 cells were cultured in the serum-free medium SF-SFM (SuZhou world-medium Biotechnology Co., Ltd., SuZhou, China) supplemented with 1% antibiotics (10,000 units/mL penicillin and 10,000 μg/mL streptomycin) (GEN-VIEW SCIENTIFIC INC., Jacksonville, FL, USA) at 27 °C. One day before transfection, S2 cells (1.0 × 10^6^ cells/well) were seeded into 6-well plates. The three expression vectors (4 µg) were co-transfected with the pCoHygro selection vector (0.2 µg) into S2 cells using Cellfectin II Reagent (ThermoFisher, Waltham, MA, USA), according to the manufacturer′s instructions. Hygromycin B (Beyotime Biotechnology, Shanghai, China) was added to the medium 24 h post-transfection, at a final concentration of 0.5 mg/mL to begin selection. After selection, four times in total, at 5-day intervals, stable integrants S2-aE2, S2-aE2Fc, and S2-aE2ΔFc were obtained. Ultimately, partial integrants were treated with CuSO_4_ at a final concentration of 0.5 mmol/L for protein expression and confirmed by western blotting.

### 2.2. Expression and Purification of APPV E2, E2Fc, and E2ΔFc Fusion Proteins

The recombinant *Drosophila* cell lines S2-aE2, S2-aE2Fc, and S2-aE2ΔFc, were transferred to a 500 mL Erlenmeyer flask for suspension culture at 27 °C. When the cell density reached 4 × 10^6^ cells/mL, CuSO_4_ (final concentration 0.5 mmol/L) was added to induce protein expression. After 4 days of induction, 40 μL of supernatant samples were collected and subjected to western blotting. Briefly, the supernatant samples were separated via 12% sodium dodecyl sulfate polyacrylamide gel electrophoresis (SDS-PAGE) and subsequently transferred onto polyvinylidene difluoride (PVDF) membranes. The membranes were blocked with 5% skim milk in phosphate-buffered saline with Tween 20 (PBST) for 1 h at room temperature (RT). The blocked membranes were incubated with anti-His monoclonal antibody (1:5000 dilution in PBS) for 2 h at RT, washed three times with PBST, and then incubated with horseradish peroxidase (HRP)-conjugated goat anti-mouse IgG (1:5000 dilution in PBS) (MBL, Nagoya, Japan) for 1 h at RT. After intensive washing, protein bands were detected using the ECL chemiluminescence system (UElandy Inc., SuZhou, China) and analyzed using Image Lab software 4.0.1 (BIO-RAD, Hercules, CA, USA).

To purify the APPV E2, E2Fc, and E2ΔFc fusion proteins, the culture supernatants were centrifuged at 10,000 r/min for 30 min and filtered using 0.22 μm filters to remove residual cell debris. Then, the APPV E2, E2Fc, and E2ΔFc fusion proteins were purified using the NGC Quest 10 chromatography system (BIO-RAD, Hercules, CA, USA) through a nickel affinity chromatography column (GE Healthcare, Shanghai, China) in accordance with the manufacturer′s protocol. Purified APPV E2, E2Fc, and E2ΔFc fusion proteins were confirmed via 12% SDS-PAGE and quantified using a Pierce^TM^ BCA Protein Assay Kit (ThermoFisher, Waltham, MA, USA). The APPV E2, E2Fc, and E2ΔFc protein dimers were analyzed via SDS-PAGE and western blotting in the presence or absence of the reducing agent β-mercaptoethanol.

### 2.3. Co-localization Detection of APPV E2Fc and E2ΔFc Fusion Proteins with FcγRI

The binding of APPV E2Fc and E2ΔFc fusion proteins to Fc gamma receptor I (FcγRI) was detected by indirect immunofluorescence assay (IFA), as previously described [16]. Briefly, porcine alveolar macrophages (2.0 × 10^5^ cells/well) were seeded into 24-well plates and cultured in Roswell Park Memorial Institute (RPMI) 1640 (Gibco, Carlsbad, CA, USA) containing 20% fetal bovine serum (GEN-VIEW SCIENTIFIC INC., Jacksonville, FL, USA). APPV E2, E2Fc, or E2ΔFc protein (2 μg) was added to the wells when the cells reached 60–70% confluence. After incubation for 12 h at 37 °C, cells were washed with PBST three times and fixed with 4% paraformaldehyde in PBS for 30 min at RT, permeabilized with 0.5% Triton X-100 for 20 min at −20 °C, and further blocked with 5% BSA for 1 h at RT. Subsequently, the blocked cells were incubated with rabbit anti-FcγRI/CD64 polyclonal antibody (1:500 dilution in PBS) (Shanghai Xin Yu Biotech Co., Ltd., Shanghai, China) and APPV E2 monoclonal antibody (mAb) (1:1000 dilution in PBS) (a gift from Dr. Huawei Zhang, College of Veterinary Medicine, Huazhong Agricultural University) for 2 h at 37 °C. Then, the cells were washed with PBST three times and incubated with Alexa Fluor 488 goat anti-rabbit antibody (1:1000 dilution in PBS) and Alexa Fluor 555 goat anti-mouse antibody (1:1000 dilution in PBS) (ThermoFisher, Waltham, MA, USA) for 1 h at 37 °C. After intensive washing, the cells were analyzed using a laser scanning confocal microscope (LSM 510, Zeiss, Thornwood, NY, USA).

### 2.4. Antigenicity and Affinity Studies of APPV E2, E2Fc, and E2ΔFc Fusion Proteins

To detect the antigenicity of APPV E2, E2Fc, and E2ΔFc fusion proteins, 96-well flat-bottomed plates were pre-coated overnight at 4 °C with 50 μL of 2 μg/mL purified APPV E2, E2Fc, or E2ΔFc fusion protein, and the plate was washed thrice with PBST. After blocking with 5% BSA for 1 h at RT, the plate was incubated with APPV E2 mAb or CSFV E2 mAb (1:1000 dilution in PBS) (Wuhan Keqian Biology Co., Ltd., Wuhan, China) for 90 min at 37 °C. Subsequently, the plate was washed three times with PBST and incubated with HRP-conjugated goat anti-mouse IgG (1:10,000 dilution in PBS) for 45 min at 37 °C. After intensive washing, 100 μL of enzyme substrate solution (TMB) was added to each well and incubated for 20 min at RT in the dark, and the reaction was stopped by adding 50 µL of 2 mol/L H_2_SO_4_. Finally, the absorbance was measured at 450 nm wavelength.

Detection of the binding affinity of APPV E2Fc and E2ΔFc fusion proteins to FcγRI on APCs was performed following a protocol similar to that described above, except that the 96-well plates were pre-seeded with porcine alveolar macrophages (5 × 10^4^ cells/well), followed by sequential incubation with serially diluted purified APPV E2, E2Fc, or E2ΔFc fusion protein and anti-His mAb (1:5000 dilution in PBS) and HRP-conjugated goat anti-mouse IgG (1:10,000 dilution in PBS).

### 2.5. Vaccination of Piglets

Animal experiments were performed in accordance with protocols approved by the Animal Ethical and Welfare Committee of the College of Veterinary Medicine, Huazhong Agricultural University, Hubei, China (No.00288687). The purified APPV E2, E2Fc, or E2ΔFc protein was emulsified with ISA 201VG adjuvant at a ratio of 1:1 (*w*/*ο*/*w*) or IMS 1313VG adjuvant (Seppic, Paris, France) at a ratio of 1:1 (*w*/*w*) according to the manufacturer′s instructions. A total of 33 7-week-old weaned piglets were purchased from the experimental farm of Huazhong Agricultural University. The piglets were confirmed to be seronegative for APPV by indirect ELISA based on the E^rns^ protein, as previously described [25]. The piglets were randomly divided into six vaccine-immunized groups (five piglets per group) and one PBS control group (three piglets). The detailed immunization procedures are listed in Table 1. All piglets were injected with the same dose of subunit vaccines for booster immunization 14 days post-primary immunization (dpi). Serum samples were collected at 0, 14, 28, and 42 dpi and stored at −20 °C until use.

### 2.6. Detection of APPV E2-Specific Antibodies

An indirect ELISA based on E2 protein was performed to detect specific antibodies elicited by APPV E2, E2Fc, or E2ΔFc protein immunization, as shown in a previous study [26]. The purified APPV E2 protein (2 μg/mL) was coated onto a 96-well flat-bottomed plate overnight at 4 °C, and then the plate was washed with PBST three times. After blocking with 5% BSA for 1 h at RT, the plate was incubated with serum samples (1:32,000 dilution in PBS) for 90 min at 37 °C. Subsequently, the plate was washed three times with PBST and incubated with HRP-conjugated goat anti-swine IgG (1:10,000 dilution in PBS) (AntGene, Wuhan, China) for 45 min at 37 °C. After intensive washing, 100 μL of enzyme substrate solution (TMB) was added to each well and incubated for 20 min at RT in the dark, and the reaction was stopped by adding 50 µL of 2 mol/L H_2_SO_4_. Finally, the absorbance was measured at 450 nm wavelength.

### 2.7. Lymphocyte Proliferation Assay

To evaluate the proliferation of T lymphocytes, whole blood (10 mL) was collected from immunized piglets in each group at 42 dpi, and peripheral blood lymphocytes were isolated using a pig peripheral blood lymphocyte isolation kit (TBDsciences, Tianjin, China) in accordance with the manufacturer’s protocol. The lymphocyte proliferation assay was performed as described in our previous study [14]. Briefly, lymphocytes (4 × 10^6^ cells/mL) were seeded into a 96-well plate with 100 µL RPMI-1640 containing 20% FBS and stimulated with purified APPV E2 protein (10 µg/mL), concanavalin A (10 µg/mL), and 100 µL RPMI-1640 containing 20% FBS. After 72 h of culture at 37 °C, 10 µL CCK-8 reagent (MedChemExpress, Shanghai, China) was added to each well and incubated at 37 °C for 4 h. Finally, the absorbance was measured at 450 nm wavelength, and the stimulation index (SI) was calculated according to the formula: SI = (OD values of immunized groups − OD values of blank control)/(OD values of negative control − OD values of blank control).

### 2.8. Detection of Cytokines

Peripheral blood lymphocytes were isolated as described previously. Lymphocytes (4 × 10^6^ cells/mL) were seeded into a 24-well plate and stimulated with purified APPV E2 protein (10 µg/mL). After 48 h of incubation at 37 °C, the culture supernatants were collected and determined using commercially available porcine interferon (IFN)-γ, interleukin (IL)-2, interleukin (IL)-4, and interleukin (IL)-10 ELISA kit (Neobioscience, Shenzhen, China) following the manufacturer′s protocol. The absorbance was measured at 450 nm wavelength, and the concentrations of the different cytokines were calculated using a standard curve.

### 2.9. Statistical Analysis

All data were analyzed using GraphPad Prism 8.0 software (GraphPad Software Inc., La Jolla, CA, USA) and represented as the mean ± standard deviation (SD). Comparisons were performed using one- or two-way ANOVA followed by Tukey’s test. Statistical significance was set at *p* < 0.05.

## 3. Results

### 3.1. Construction of Recombinant Drosophila Cell Lines

**Figure 1:**
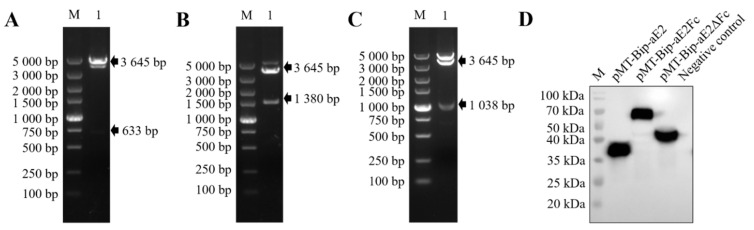
Identification of the recombinant *Drosophila* cell lines. The recombinant plasmids pMT-Bip-aE2 (**A**), pMT-Bip-aE2Fc (**B**), and pMT-Bip-aE2ΔFc (**C**) were digested with *EcoR* I and *Xho* I and analyzed via 1% agarose gel electrophoresis. M, DL 5000 DNA Marker. (**D**) Western blotting analysis of APPV E2, E2Fc, and E2ΔFc proteins expressed in S2 cells. The pMT-Bip-V5-HisA vector was used as a negative control. The S2 cell lines, which stably express APPV E2, E2Fc, and E2ΔFc proteins, were induced by CuSO_4_ and cultured at 27 °C for 4 days. M, protein marker.

The pMT-Bip-V5-HisA vectors expressing APPV E2, E2Fc, and E2ΔFc fusion proteins were constructed. The expression vectors were identified with *EcoR* I/*Xho* I restriction enzyme and analyzed via 1% agarose gel electrophoresis, and three specific bands of approximately 633 bp, 1380 bp, and 1038 bp were observed (Figure 1A–C). Nucleotide sequencing showed that the expression vectors pMT-Bip-aE2, pMT-Bip-aE2Fc, and pMT-Bip-aE2ΔFc were successfully constructed.

The supernatants derived from S2 cells were analyzed via western blotting using anti-His monoclonal antibodies. Three single protein bands of approximately 36 kDa, 70 kDa, and 48 kDa were observed (Figure 1D), confirming that the stable integrants S2-aE2, S2-aE2Fc, and S2-aE2ΔFc were successfully constructed.

### 3.2. Purification of APPV E2, E2Fc, and E2ΔFc Fusion Proteins

**Figure 2:**
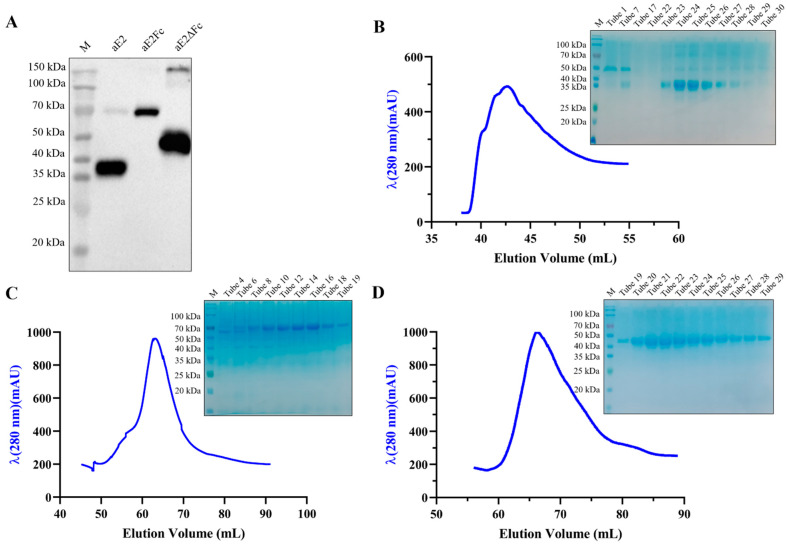
Purification of APPV E2, E2Fc, and E2ΔFc fusion proteins from S2 cells. (**A**) Western blotting analysis of APPV E2, E2Fc, and E2ΔFc proteins before purification. Elution peak curve of APPV E2 (**B**), E2Fc (**C**), and E2ΔFc (**D**) fusion proteins. The SDS-PAGE profiles were shown in the upper right corner of the elution curve. The S2 cell lines, which stably express APPV E2, E2Fc, and E2ΔFc proteins, were induced by CuSO_4_ and cultured at 27 °C for 4 days. M, protein marker.

To prepare the purified APPV E2, E2Fc, and E2ΔFc fusion proteins, the recombinant S2 cell lines S2-aE2, S2-aE2Fc, and S2-aE2ΔFc were transferred to a 500 mL Erlenmeyer flask for suspension culture and induced by CuSO_4_. After 4 days of induction, 40 μL supernatant samples were collected and subjected to western blotting to confirm the presence of APPV E2, E2Fc, and E2ΔFc fusion proteins (Figure 2A). Then, the supernatants containing APPV E2, E2Fc, and E2ΔFc fusion proteins were harvested by centrifugation and purified by Ni-NTA chromatography. Based on the UV absorption peak value of the NGC Quest 10 chromatography system, APPV E2, E2Fc, and E2ΔFc fusion proteins were eluted with 80 mM imidazole, and finally analyzed via SDS-PAGE (Figure 2B–D).

### 3.3. Characterization of APPV E2, E2Fc, and E2ΔFc Dimer

**Figure 3:**
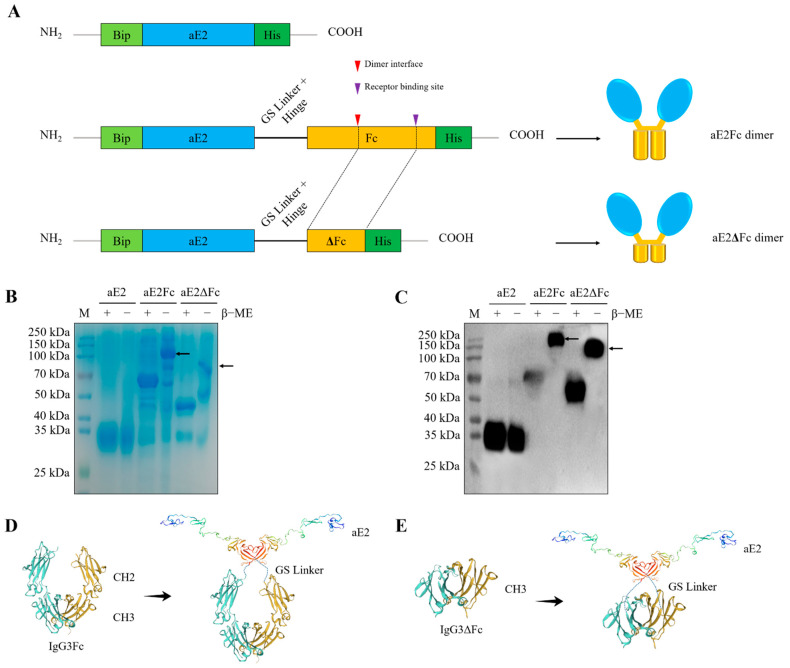
Characterization of APPV E2, E2Fc, and E2ΔFc fusion proteins. (**A**) Schematic design of APPV E2, E2Fc, and E2ΔFc proteins. APPV E2, E2Fc, and E2ΔFc recombinant proteins were analyzed via SDS-PAGE (**B**) and western blotting with a monoclonal antibody against APPV E2 (**C**) in the presence (+) or absence (−) of the reducing agent β-mercaptoethanol. M, Marker; β-ME, β-mercaptoethanol. The black arrow indicates the bands of the Fc or ΔFc stabilized dimer. Structures of APPV E2Fc (**D**) and E2ΔFc (**E**) proteins. The models were built using the SWISS-MODEL server (https://swissmodel.expasy.org/interactive, accessed on 2 June 2021), with SMTL ID: 2yq2.1.A (aE2), 5w38.1.A (IgG3Fc), and 5hsf.1.A (IgG3ΔFc) used as the templates.

The Fc fusion protein can form a stable dimer through the disulfide bond of the Fc hinge region (Figure 3A), which increases the stability and half-life of the protein [21,22,23,24]. To investigate whether E2Fc and E2ΔFc fusion proteins can form dimers, the APPV E2, E2Fc, and E2ΔFc fusion proteins were analyzed by SDS-PAGE and western blotting in the presence or absence of the reducing agent β-mercaptoethanol. As shown in Figure 3B,C, the molecular weights of the E2Fc protein (~140 kDa) and E2ΔFc protein (~96 kDa) under non-denaturing conditions was twice that of denaturing conditions, while the molecular weight of E2 protein (~36 kDa) did not change. These results suggest that E2Fc and E2ΔFc, but not E2, can form dimers. Structures of APPV E2Fc and E2ΔFc proteins were also simulated using the SWISS-MODEL server (https://swissmodel.expasy.org/interactive (accessed on 2 June 2021) (Figure 3D,E).

### 3.4. Binding of APPV E2Fc and E2ΔFc Fusion Proteins to FcγRI on APCs

**Figure 4:**
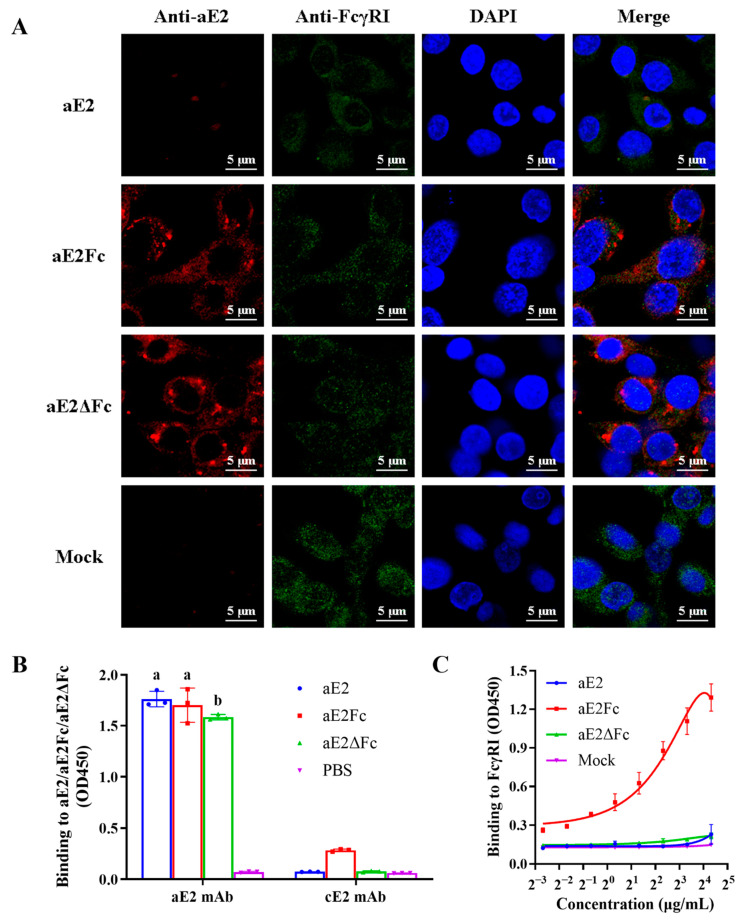
Functionality and antigenicity of APPV E2, E2Fc, or E2ΔFc fusion proteins. (**A**) Co-localization of FcγRI on macrophages and APPV E2Fc or E2ΔFc fusion protein. Immunofluorescent microscopic photomicrographs displayed localization of FcγRI on macrophages and the pAb FcγRI (CD64)-immunoreactive green fluorescence was detected by Alexa Fluor 488 goat anti-rabbit antibody. While the localization of E2 in FcγRI was analyzed through the mAb E2-immunoreactive red fluorescence using Alexa Fluor 555 goat anti-mouse antibody. The nuclei (blue) were labeled with DAPI. Binding affinity of APPV E2, E2Fc, and E2ΔFc fusion proteins to APPV-specific (aE2) mAb (**B**) and FcγRI (**C**). ELISA was performed to detect the binding affinity of APPV E2, E2Fc, and E2ΔFc fusion proteins to APPV-specific (aE2) mAb and FcγRI. Binding affinity was characterized as OD450 value. CSFV-specific (cE2) mAb was assessed to investigate the cross-reactivity. PBS was used as a negative control. Different letters (a, b) indicate a statistically significant difference between different experimental groups (*p* < 0.05). Experiments were conducted independently in triplicates. Data are represented as the mean ± SD (n = 3).

To determine whether the Fc fusion protein can increase its ability to bind to its receptor FcγRI, we identified the binding of APPV E2Fc and E2ΔFc fusion proteins to FcγRI on antigen-presenting cells (APCs) through IFA. The results demonstrated that APPV E2Fc and E2ΔFc fusion proteins had a strong binding ability to FcγRI on APC compared with E2 protein, while the intracellular accumulation of protein suggests uptake by Fc-mediated phagocytosis (Figure 4A).

### 3.5. Antigenicity and Affinity Studies of APPV E2, E2Fc, and E2ΔFc Fusion Proteins

The antigenicity of APPV E2, E2Fc, and E2ΔFc fusion proteins was determined using ELISA to test their binding to APPV-specific (aE2) mAb. Meanwhile, CSFV-specific (cE2) mAb was assessed to investigate cross-reactivity. The results revealed that the three recombinant proteins expressed in S2 cells bound strongly to APPV-specific (aE2) mAb but did not cross-react with CSFV-specific (cE2) mAb (Figure 4B), confirming their antigenicity. ELISA analysis demonstrated a dose-dependent affinity between APPV E2Fc fusion protein and FcγRI, while no affinity was observed between APPV E2ΔFc or APPV E2 and FcγRI (Figure 4C).

### 3.6. APPV E2Fc or E2ΔFc Immunization Induce an Efficient Antibody Response in Piglets

**Figure 5:**
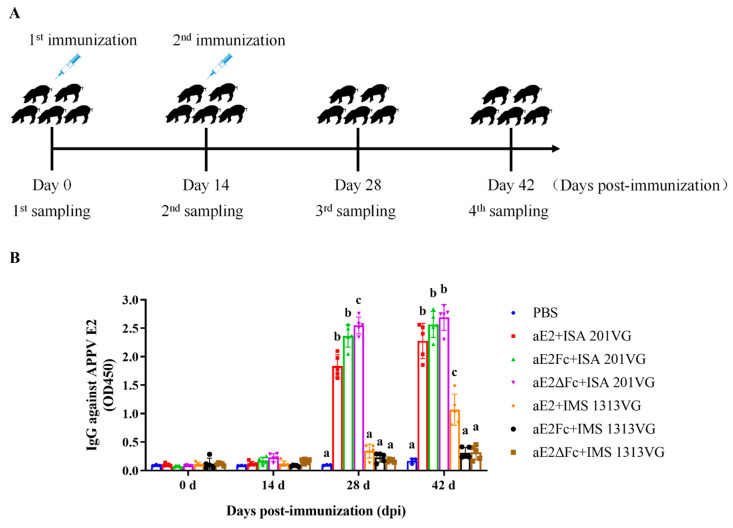
Detection of APPV E2-specific antibodies. (**A**) Schematic outline of the experimental design. (**B**) APPV E2-specific IgG detected via indirect ELISA at indicated dpi. Different letters (a, b, c) indicate a statistically significant difference between different experimental groups (*p* < 0.05). Experiments were conducted independently in triplicates. Data are represented as the mean ± SD (n = 5).

To assess whether E2Fc or E2ΔFc subunit vaccines can effectively promote the humoral immune response in piglets, APPV E2-specific antibodies were detected by indirect ELISA. A schematic outline of the experimental design is shown in Figure 5A. As shown in Figure 5B, at 28 dpi, the serum antibody levels of the ISA 201VG immunized groups were significantly higher than those of the IMS 1313VG (*p* < 0.05), and the serum antibody level of the aE2ΔFc immunized group was significantly higher than that of the aE2Fc and aE2 groups (*p* < 0.05). In addition, there was no significant difference between the IMS 1313VG and PBS groups (*p* > 0.05), indicating that APPV E2-specific antibodies were not induced in IMS 1313VG groups at this time. At 42 dpi, the serum antibody level reached the highest value, and the serum antibody level of the aE2ΔFc immunized group was slightly higher than that of the aE2Fc and aE2 groups (*p* > 0.05). In the IMS 1313VG adjuvant groups, only the aE2 group showed a slight increase in antibody levels, but it was still significantly lower than that of the ISA 201VG group (*p* < 0.05). These results suggested that the vaccines prepared with the ISA 201VG adjuvant could induce a stronger humoral immune response than the IMS 1313VG adjuvant. Additionally, the aE2ΔFc fusion protein can be used as a promising vaccine candidate to prevent APPV infection.

### 3.7. Lymphocyte Proliferative Response

**Figure 6:**
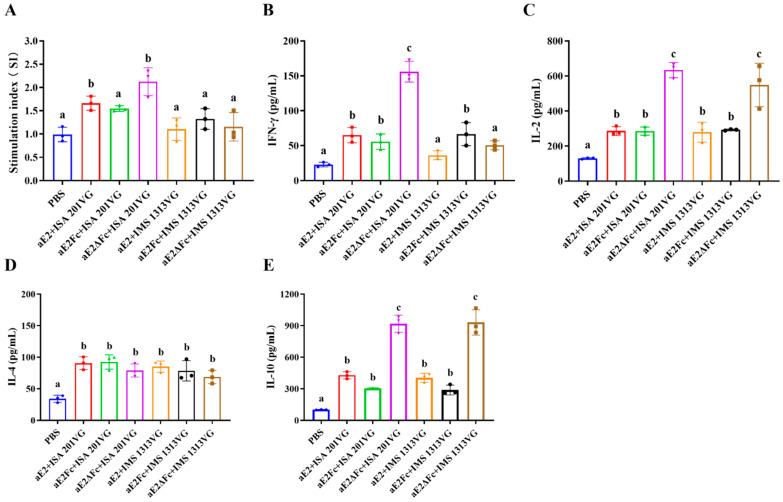
Lymphocyte proliferation assay and detection of cytokines. (**A**) Detection of lymphocyte proliferative responses. (**B**–**E**) Detection of the levels of cytokines in the supernatants of stimulated lymphocytes from piglets immunized with the APPV E2 subunit vaccines. Different letters (a, b, c) indicate a statistically significant difference between different experimental groups (*p* < 0.05). Experiments were conducted independently in triplicates. Data are represented as the mean ± SD (n = 3).

A lymphocyte proliferation assay was performed to evaluate the proliferative responses of APPV E2-specific lymphocytes. As shown in Figure 6A, the ISA 201VG immunized groups elicited proliferative responses. Furthermore, the aE2ΔFc+ISA 201VG immunized group showed the highest SI compared with the other groups. However, there was no significant difference between the IMS 1313VG and PBS groups (*p* > 0.05). These results indicated that the vaccines prepared with the ISA 201VG adjuvant significantly promoted the lymphocyte proliferative response.

### 3.8. APPV E2ΔFc Immunization Improves the Th2-Biased Cellular Immune Response

To analyze the cellular immune response in the piglets vaccinated with APPV E2, E2Fc, and E2ΔFc fusion proteins, the levels of Th1- and Th2-type cytokines were measured by the double antibody sandwich ELISA. As shown in Figure 6B–E, IFN-γ, IL-2 (Th1-type cytokine) and IL-4, IL-10 (Th2-type cytokine) levels in all ISA 201VG immunized groups were significantly higher than those of the negative control groups (*p* < 0.05). In the IMS 1313VG immunized groups, only the levels of IL-4 and IL-10 were significantly higher than those of the negative control groups (*p* < 0.05), while the levels of IFN-γ and IL-2 in the aE2+IMS 1313VG immunized groups were not significantly different from those in the negative control groups (*p* > 0.05). Notably, the levels of IL-10 in the aE2ΔFc immunized groups were markedly higher than those of other cytokines (*p* < 0.05), implying that immunization with APPV E2ΔFc improves the Th2-biased cellular immune response.

## 4. Discussion

In this study, recombinant *Drosophila* cell lines expressing APPV E2, E2Fc, and E2ΔFc fusion proteins were successfully constructed. APPV E2Fc and E2ΔFc fusion proteins purified from recombinant cell lines were demonstrated to form stable dimers. E2Fc and E2ΔFc fusion proteins could bind to FcγRI on APCs, and the affinity of E2Fc to FcγRI was higher than that of E2ΔFc. Moreover, subunit vaccines based on these three proteins were prepared, and their immunogenicity was evaluated in piglets. The results showed that the ISA 201VG adjuvant emulsified vaccines elicited a stronger immune response than the IMS 1313VG adjuvant, with aE2ΔFc+ISA 201VG having the best immune response. Therefore, we concluded that the APPV E2 subunit vaccine fused with the Fc fragments may be a promising vaccine candidate against APPV.

CT type A-II of piglets has widely emerged in swine herds, resulting in huge economic losses to the pig industry worldwide [2,3,7,17,18,19,20,27,28]. Accumulating evidence indicates that CT type A-II is associated with APPV; however, as a newly emerging virus, there are no commercial vaccines or antiviral drugs against APPV infection. Traditional vaccines, including inactivated and live attenuated vaccines, play a certain role in the clinical control of infectious diseases. However, their efficacy and safety are not guaranteed. The development of traditional vaccines is usually restricted by the characteristics of the virus itself, the most critical of which is the need to isolate bioactive virions from cell cultures. Moreover, the difficulty in producing APPV particles in cell cultures substantially hampers the development of APPV vaccines. Therefore, it is urgent to develop a novel and efficient vaccine to prevent and control APPV infections.

The E2 protein of pestiviruses plays a crucial role in the viral life cycle [29,30,31]. Numerous studies have shown that the E2 protein is the major immunogen of pestiviruses. In our previous study, we confirmed that the APPV E2 subunit vaccine induced a Th2-type immune response in mice [14]. A recent study showed that virus-like particles based on the self-assembly of E^rns^ and E2 proteins could induce a strong antibody response and reduce the viral load in the tissues of BALB/c mice [15]. These studies suggest that E2 protein can be developed as a safe and effective subunit vaccine against APPV infection, but its immunogenicity in pigs remains to be further explored.

The IgGFc region exhibits robust immunological functions by binding to Fc receptors (FcRs). Previous studies have shown that therapeutic antibodies and Fc fusion proteins interact with neonatal FcR (FcRn) to extend their serum half-life, thereby reducing the dosage and frequency of administration, and significantly improving its efficacy [21,22,23,32,33]. Fc fusion proteins display excellent potential for use in vaccine development. In our previous study, the swine IgG3Fc fragment was selected to construct the CSFV E2Fc fusion protein, which increased the immune response in piglets through the presentation of FcγRI and provided complete protection against CSFV Shimen strain [16]. Similarly, in this study, the swine IgG3Fc fragment was selected to construct the APPV E2Fc fusion protein, which was expressed and purified in S2 cells. To minimize the influence of exogenous sequences on E2 immunogenicity, we further truncated the Fc fragment, retaining only the dimer interface and receptor binding sites, and expressed and purified the APPV E2ΔFc fusion protein in S2 cells. We also constructed APPV E2 protein as a control. The three recombinant proteins were characterized and evaluated, and the results showed that aE2Fc and aE2ΔFc fusion proteins could form stable dimers, and that the prepared subunit vaccines could induce stronger humoral and cellular immune responses in piglets. Previous studies have indicated that the Fc fusion protein facilitates the dimerization of the protein through disulfide bonds in the Fc hinge region, increasing the stability and half-life of the protein [21,22,23,24]. We also demonstrated that aE2Fc and aE2ΔFc fusion proteins bind to FcγRI on APCs and enhance antigen presentation, which is consistent with previous reports [16,34]. Additionally, we determined the binding affinity of the three proteins to FcγRI on APCs. The results indicated that the binding affinity of aE2Fc to FcγRI was significantly higher than that of aE2ΔFc and aE2, while aE2ΔFc was not significantly different from that of aE2 and the blank control group. A possible reason is that although the truncated Fc region retains its receptor binding sites, its higher-order structure of the FcγRI-binding region of Fc (i.e., CH2-CH3 interface) changes, which reduces the binding affinity of the aE2ΔFc fusion protein to FcγRI [32]. Interestingly, the results of the immune test showed that aE2ΔFc induced a stronger immune response in piglets, suggesting that in addition to being presented by binding to FcRs, the Fc fusion protein may also reach T cells through other mechanisms, thus increasing the adaptive immune response. However, the specific mechanism requires further exploration.

The main obstacle in the development of APPV vaccines is that there is no suitable animal challenge model to evaluate the protective efficacy of the vaccine. Although some researchers have successfully reproduced the animal challenge model of APPV by inoculating APPV-positive serum in the early- or mid-gestation period of sows [2,3], the process is complicated and costly, which is not conducive to preclinical evaluation during vaccine development. Moreover, the difficulty in producing APPV particles in cell cultures limits the evaluation of vaccine efficacy. In particular, the level of neutralizing antibodies in the serum after immunization cannot be determined. It may be a good choice to use pseudoviruses instead of live viruses for neutralization tests to determine serum neutralizing antibody levels since it has been well applied in the research of other viruses such as SARS-CoV-2 in recent years [35,36,37]. At the time of revising this paper, work from the Zhi Cao laboratory reported that a novel APPV strain named China/HeN01/2018 was isolated and successfully propagated in embryonic porcine kidney epithelial cells (SPEV cells) [38], which will facilitate the development of neutralization tests. Overall, APPV E2Fc and E2ΔFc can be used as promising vaccine candidates to prevent APPV-induced CT in piglets.

## 5. Conclusions

Recombinant *Drosophila* cell lines expressing APPV E2, E2Fc, and E2ΔFc fusion proteins were successfully constructed in this study. The Fc fusion proteins purified from S2 cells could form stable dimers, which elicited a robust humoral and cellular immune response and a Th2-biased immune response in piglets. This study suggests that APPV E2Fc and E2ΔFc fusion proteins can be developed as safe and effective subunit vaccines against APPV infection.

## Figures and Tables

**Table 1:** Experimental design.

Experimental Group	Number of Pigs	Antigen	Dose (μg/Pig)	Adjuvant	Delivery Route
A	5	aE2	80	ISA 201VG	Intramuscular
B	5	aE2	80	IMS 1313VG	Intramuscular
C	5	aE2Fc	80	ISA 201VG	Intramuscular
D	5	aE2Fc	80	IMS 1313VG	Intramuscular
E	5	aE2ΔFc	80	ISA 201VG	Intramuscular
F	5	aE2ΔFc	80	IMS 1313VG	Intramuscular
G	3	PBS	2 mL	-	Intramuscular

## Supplementary Materials

The following are available online at https://www.mdpi.com/article/10.3390/v13122443/s1, Table S1: Primers used to amplify APPV E2, E2Fc, and E2ΔFc segments in this study.
