# Supplementary material for: Fc-Mediated E2-Dimer Subunit Vaccines of Atypical Porcine Pestivirus Induce Efficient Humoral and Cellular Immune Responses in Piglets
Source: Viruses. 2021 Dec 6;13(12):2443. doi: 10.3390/v13122443 (PMC8703287; doi:10.3390/v13122443)
Supplement: Supplementary file 1 [file viruses-13-02443-s001.zip › viruses-1437209-supplementary.pdf]

Supplementary Materials

Fc-Mediated E2-Dimer Subunit Vaccines of Atypical Porcine Pestivirus Induce Efficient Humoral and Cellular Immune Responses in Piglets

Xujiao Ren [1,2], Ping Qian [1,2,3], Shudan Liu [1,2], Huanchun Chen [1,2,3] and Xiangmin Li [1,2,3,*]

1 State Key Laboratory of Agricultural Microbiology, Huazhong Agricultural University, Wuhan 430070, China; renxujiao92@163.com (X.R.); qianp@mail.hzau.edu.cn (P.Q.); vet2439134082@163.com (S.L.); chenhch@mail.hzau.edu.cn (H.C.)

2 Laboratory of Animal Virology, College of Veterinary Medicine, Huazhong Agricultural University, Wuhan 430070, China

3 Key Laboratory of Preventive Veterinary Medicine in Hubei Province, The Cooperative Innovation Center for Sustainable Pig Production, Wuhan 430070, China

* Correspondence: lixiangmin@mail.hzau.edu.cn; Tel.: +86-27-87282608

Table S1. Primers used to amplify APPV E2, E2Fc, and E2ΔFc segments in this study.

Primers	Sequence (5′ to 3′)	Purpose
pMT-aE2-F	GTGGAATTC[a]TTCATGCCACAAAAGAG	Amplify aE2 gene flanked with *EcoR* I and *Xho* I, and containing 6×His tag
pMT-aE2-R	GACCTCGAG[b]TCA**GTGATGGTGATGGTGATG**[c]*AGAGCCCGATC*[d]CATGACGGTACATCGTTG	
aE2-Fc′-R	GAGCCACCTCCTCCGGACCCACCCCCGCCTGATCCATGACGGTACATCGTTGCAG	Amplify fragment aE2-Fc′ containing the 5′ end of IgG3Fc
aE2′-Fc-F	TGCCTACCGTCTCATCCGCTGCAACGATGTACCGTCATGGATCAGGCGGGGGTGGGTC	Amplify fragments aE2′-Fc and aE2′-ΔFc containing the 3′ end of aE2 gene
pMT-Fc-R	GACCTCGAG[b]TCA**GTGATGGTGATGGTGATG**[c]*AGAGCCCGATCC*[d]TTTACCCTGAGTCTTGG	Amplify fragments aE2Fc and aE2ΔFc flanked with *EcoR* I and *Xho* I, and containing 6×His tag
pCAG-aE2-F	AAAGAATTC[a]TCGTGCCACAAAAGACAAGAC	Amplify the full-length glycoprotein E2 gene flanked with *EcoR* I and *Not* I, and containing 6×His tag
pCAG-aE2-R	GTAGCGGCCGC[e]CTA**GTGATGGTGATGGTGATG**[c]*AGAGCCCGATCC*[d]ACTAGCTTCCACCTG	

a: Restriction site for *EcoR* I.

b: Restriction site for *Xho* I.

c: 6×His tag sequences in bold.

d: GS Linker sequences are shown in italic.

e: Restriction site for *Not* I.
